# Collapse of a lipid-coated nanobubble and subsequent liposome formation

**DOI:** 10.1038/srep28164

**Published:** 2016-06-16

**Authors:** Kenichiro Koshiyama, Shigeo Wada

**Affiliations:** 1 Graduate School of Engineering Science, Osaka University, Toyonaka 560-8531, Japan

## Abstract

We investigate the collapse of a lipid-coated nanobubble and subsequent formation of a lipid vesicle by coarse grained molecular dynamics simulations. A spherical nanobubble coated with a phospholipid monolayer in water is a model of an aqueous dispersion of phospholipids under negative pressure during sonication. When subjected to a positive pressure, the bubble shape deforms into an irregular spherical shape and the monolayer starts to buckle and fold locally. The local folds grow rapidly in multiple directions and forming a discoidal membrane with folds of various amplitudes. Folds of small amplitude disappear in due course and the membrane develops into a unilamellar vesicle via a bowl shape. Folds with large amplitude develop into a bowl shape and a multivesicular shape forms. The membrane shape due to bubble collapse can be an important factor governing the vesicular shape during sonication.

Liposomes are spherical vesicles with lipid-bilayer membranes that enclose an aqueous space. They are being extensively used in various applications, such as models of biological cells and as carriers to deliver dietary, cosmetic, or pharmaceutical components to the body^1^,^2^. To manipulate liposome characteristics in different applications, various methods have been developed^3^. Ultrasound irradiation, i.e., sonication, is one conventional physical method used for controlling liposome sizes in the nanometer range^4^,^5^,^6^,^7^,^8^,^9^.

In sonicated water, the ultrasound waves induce bubble nucleation, bubble growth, and bubble oscillation. A phenomenon known as acoustic cavitation causes various mechanical effects, e.g., radiation force, microstreaming, microjets, and shock waves, depending on the bubble and ultrasound conditions^10^,^11^. Acoustic cavitation is widely known to be responsible for many of the biophysical effects of ultrasound on cells^12^,^13^. Liposome formation by sonication is generally performed under conditions designed to induce strong acoustic cavitation. Cavitation bubbles in the aqueous solution can be observed during the procedure and acoustic cavitation is believed to affect the formation of liposomes^6^ even though the precise control of acoustic cavitation is challenging^7^,^8^,^9^,^14^.

To date, researchers have investigated the mechanisms of liposome formation using phenomenological models of lipid assemblies^4^,^5^. It is widely accepted that liposomes form in a two-stage process: the formation of a discoidal lipid-membrane and the closure of the membrane into closed shells. Computer simulations of lipid systems such as atomistic molecular dynamics (MD), coarse-grained molecular dynamics (CGMD), and dissipative particle dynamics simulations are suitable methods to study lipid molecular dynamics in aqueous dispersions^15^. Using simulations, researchers have revealed the molecular mechanisms of liposome formation from randomly distributed lipids in water and the fusion of liposomes^16^,^17^,^18^,^19^. Furthermore, researchers have started to investigate the destruction of lipid assemblies during sonication using molecular simulations^20^,^21^,^22^,^23^. However, the effects of cavitation bubbles on liposome formation at the molecular scale have not been studied sufficiently. Recent applications of liposomes involving ultrasound have increased the demand for delicate control of liposome characteristics;^1^,^2^,^24^ therefore, it is essential to understand lipid dynamics at the molecular scale during sonication.

In this study, we investigate liposome formation in an aqueous dispersion of phospholipids during sonication using CGMD simulations of lipid–water systems. On the basis of acoustic cavitation, we focus on the lipid molecular dynamics after the formation of a single bubble via sonication and present a scenario for liposome formation as follows: (i) the negative pressure of the ultrasound wave generates or grows a water vapor bubble; (ii) the liquid–vapor interface is spontaneously coated by lipid molecules which are surface active;^25^ (iii) when subjected to a positive pressure, the bubble shrinks and the interface is fully coated by a lipid monolayer; and (iv) the lipid-coated bubble collapses and develops into a liposome. To address the essential lipid dynamics while considering the computational efficiency, the initial structure for our simulations is modeled as a preformed nanobubble coated with a lipid monolayer in water. We verify the above scenario by analyzing the collapse of the lipid-coated nanobubble and the subsequent formation of a liposome.

## Results

### Nanobubble collapse and liposome formation

**Figure 1:**
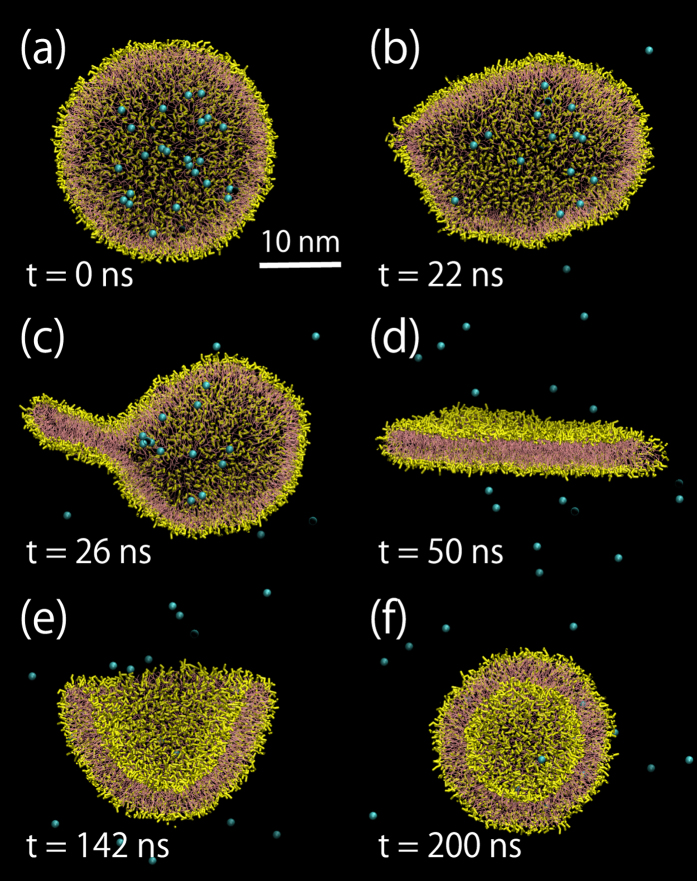
Cutaways of the lipid assembly during the collapse of a lipid nanobubble and the vesicle formation for the 2400 DPPC lipid system. The spherical bubble shape (**a**) deforms into an irregular prolate ellipsoidal shape (**b**). The monolayer buckles and folds (**b,c**) and a discoidal membrane forms (**d**). The discoidal membrane organizes into a unilamellar vesicle (**f** ) via a bowl shape (**e**). The hydrophilic headgroups are shown in yellow and the hydrophobic chains in pink. Water beads that are initially in the bubble are shown in cyan and the other water beads are not shown.

Spontaneous liposome formation from a nanobubble coated with a dipalmitoylphosphatidylcholine (DPPC) monolayer, as shown in Fig. 1 (see also the movie and Fig. S1 in the Supplementary Information), is the first numerical demonstration. When the spherical bubble is subjected to a positive pressure, its shape deforms into an irregular prolate ellipsoidal shape (Fig. 1(a,b)). The monolayer starts to buckle and fold from an apsis of the ellipsoidal bubble (Fig. 1(b,c)). The local fold grows rapidly, thereby pushing out the evaporated water beads (Fig. S1). Consequently, a discoidal membrane forms (Fig. 1(c,d)). The discoidal membrane organizes into a unilamellar vesicle via a bowl shape (Fig. 1(e,f)). Vesicle formation via discoidal and bowl shapes can be confirmed in other computer simulations of lipid systems or in the conventional theory^4^,^5^,^16^,^18^,^19^,^26^.

**Figure 2:**
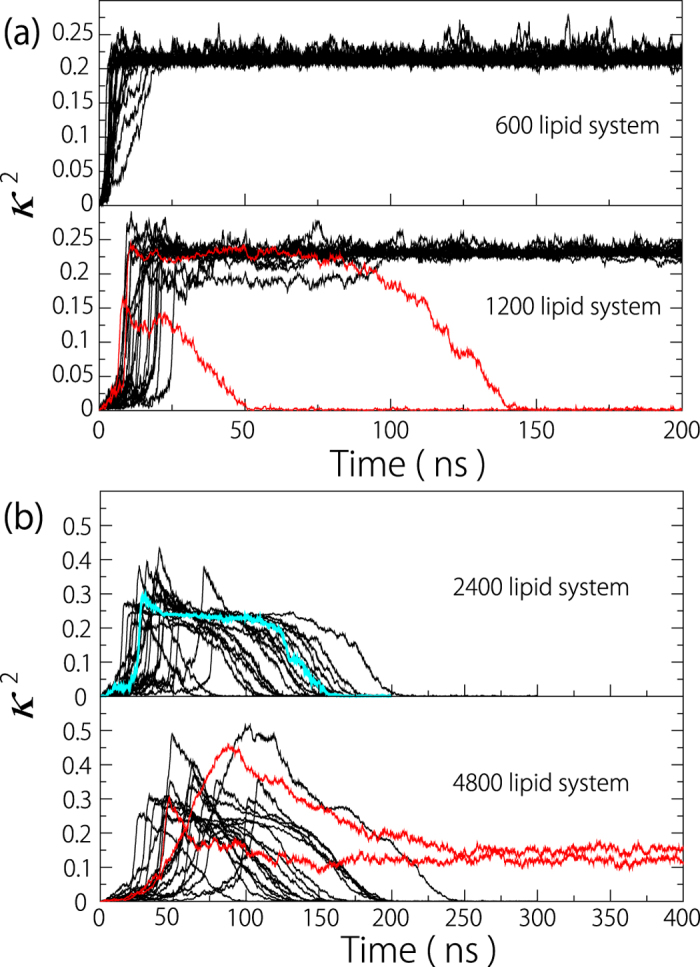
Temporal evolution of the relative shape anisotropy *κ*^2^ of 20 samples for the 600 and 1200 lipid systems (**a**) and for the 2400 and 4800 lipid systems (**b**). In (**a**), the red lines show the results of the transition to a vesicular shape. In (**b**), the cyan line shows the temporal evolution in Fig. 1 and the red lines show the results of the transition to a multivesicular shape.

As a quantitative indication of the liposome formation process shown in Fig. 1, we analyzed the instantaneous relative shape anisotropy of a lipid assembly *κ*^2^, which is defined using the invariants of the instantaneous gyration tensor^18^,^27^,^28^. The cyan line in Fig. 2(b) shows the temporal evolution of *κ*^2^ in Fig. 1. As expected, we can confirm the shape change history of the lipid assembly by *κ*^2^; *κ*^2^ starts from zero (spherical shell), suddenly increases at approximately 25 ns (monolayer collapse), stays at approximately 0.25 (discoidal membrane), and finally recovers to zero (unilamellar vesicle).

### Effect of the number of lipids

The process of liposome formation depends on the number of lipids coating the bubble (Fig. 2). For the 600 lipid system, all values of *κ*^2^ remain approximately 0.25 after the stepwise increase, showing no vesicle formation after the bubble collapse. For the 1200 lipid system, *κ*^2^ occasionally recovers to zero (denoted by red lines in Fig. 2 (a)), indicating the transition to a vesicular shape (10% of the total samples). For the 2400 lipid system, all values of *κ*^2^ recover to zero, and all the resultant vesicular shapes have been confirmed to become unilamellar vesicles (data not shown). This could be explained by the balance between edge and bending energies of the lipid assembly^4^,^5^, although it is essentially statistical at the molecular scale.

**Figure 3:**
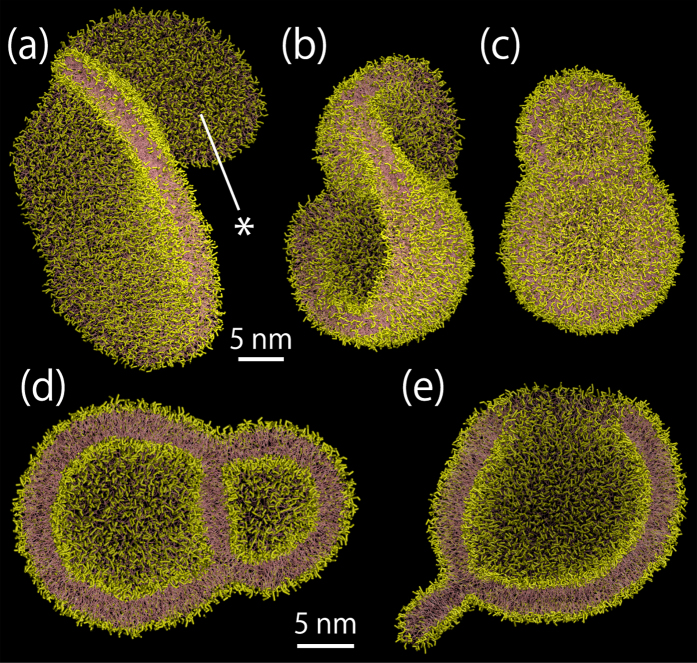
Snapshots of the double vesicular formation (**a–c**), the cutaways of the double vesicular shape (**d**) and of the intermediate bowl shape with a fold (**e**). The cutaways are enlarged for clarity. The asterisk in (**a**) indicates a fold with large amplitude.

Interestingly, for the 4800 lipid system, 10% of the total samples do not recover to zero and stay in the range from 0.1 to 0.2 (shown by red lines in Fig. 2(b)). Figure 3 shows the representative process when *κ*^2^ does not recover. An ear-like lipid assembly, i.e., a fold with large amplitude (indicated by an asterisk in Fig. 3(a)), forms after the bubble collapse; the fold and the primary discoidal membrane develop into a bowl shape(Fig. 3(b)) and form a double-vesicular shape (Fig. 3(c,d)). The similar vesicular structure has been reported as the hemifusion diaphragm in a simulation study on vesicle fusion^17^. We extended the relaxation simulation, but the double-vesicular shape was stable for at least 1 µs.

### Effect of fold amplitude

Most folds that are formed immediately after the collapse (Fig. S2 in the Supplementary information) disappear in due course. For the 600 and 1200 lipid systems, the discoidal membranes have a flat shape transiently. For the 2400 and 4800 lipid systems, occasionally, the discoidal membranes develop into bowl shapes, without showing apparent flatness (Fig. 2(b), where some of *κ*^2^ do not transiently remain approximately 0.25). To investigate the effects of the intermediate discoidal membrane shape on the resultant unilamellar vesicle shape, we evaluated the ratio *R*_*io*_ of the number of lipids between inner and outer membrane leaflets of the unilamellar vesicle. The lipid vector from the geometrical center of hydrophobic beads to that of hydrophilic beads of the MARTINI lipid model was used as an indication.

**Figure 4:**
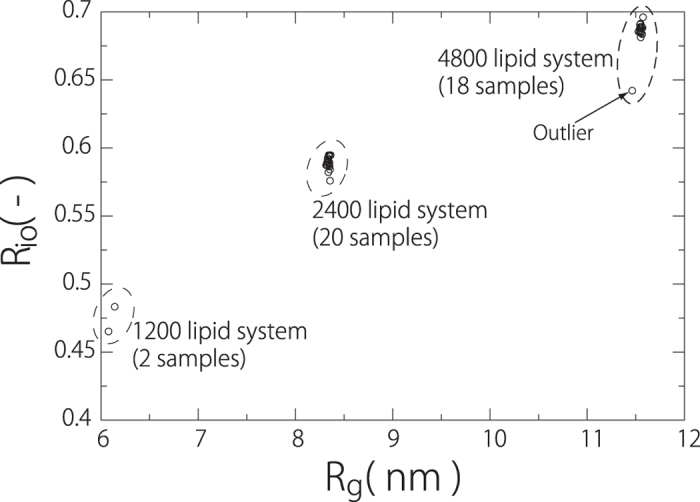
Relationship between *R*_io_, the ratio of the number of lipids between the inner and the outer membrane leaflets of the unilamellar vesicles, and *R*_g_, the radius of the gyration tensor. The arrow shows the outlier.

Figure 4 shows the relationship between *R*_*io*_ and the radius of gyration tensor *R*_*g*_ for the 1200, 2400 and 4800 lipid systems. With increasing number of lipids, *R*_*io*_ and *R*_*g*_ increase. In addition, the larger *R*_*g*_ is, the larger *R*_*io*_ will be; this can be explained by the fact that the difference between the curvatures of the two monolayers decreases. *R*_*io*_ for the 2400 lipid system agrees with the results of the MARTINI lipid vesicle obtained by different protocols (0.57)^29^. The variations in *R*_*io*_ for each lipid system are small, but there is an outlier for the 4800 lipid system. The intermediate membrane shape is shown in Fig. 3(e), where the transition from the bowl shape to the unilamellar vesicle occurs while maintaining a fold with small amplitude; this results in a larger number of lipids in the outer membrane leaflet.

### Effect of saturation of phospholipid tails

**Figure 5:**
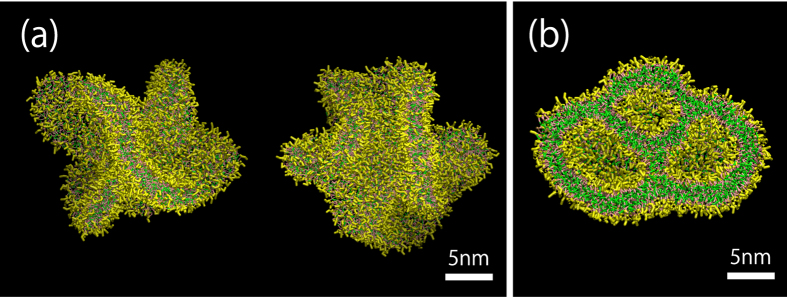
Lipid assembly just after the collapse of a nanobubble coated with an unsaturated phosphatidylc-holine monolayer for the 4800 lipid system (**a**) and the cutaway of a multivesicular shape (**b**). The snapshots from two different directions are shown in (**a**). The unsaturated tails’ beads are shown in green.

Finally, we investigate liposome formation from the nanobubble coated with an unsaturated phosphatidylcholine (dilinoleyl-PC) monolayer in an attempt to verify the impact of saturation of phospholipid tails. When we analyzed the lipid dynamics of 10 samples for the 600 dilinoelyl-PC lipid system, all values of *κ*^2^ revert to zero (Fig. S3 in the Supplementary information), unlike for the 600 DPPC lipid system (Fig. 2(a)). In addition, most of *κ*^2^ values do not transiently reach 0.25, which means the dilinoleyl-PC lipid assembly develops into a vesicle shape without showing apparent flatness. The discoidal membrane shape after the collapse is very complicated and it has many folds for the larger system (Fig. 5(a)). Consequently, this induces a transient multivesicular shape formation (Fig. 5(b)).

## Discussion

Liposome formation is simply understood as a two-stage mechanism where liposomes form via discoidal and bowl shapes^4^,^26^. To understand the mechanisms of liposome formation during sonication at the molecular scale, we investigated the lipid molecular dynamics after a single nanobubble formed in an aqueous dispersion. We showed that, associated with the bubble collapse under a positive pressure, a discoidal membrane with folds of various shapes and amplitudes forms (Figs 1 and 5 and S2). The complex membrane shape after the collapse can induce further formation of various vesicular-shapes, depending on the number of lipids and the saturation (Figs 2, 3, 4, 5). A multivesicular shape can then be observed (Figs 3 and 5). These atomistic-level observations shed light on the importance of the lipid-coated bubble collapse and the mechanism of liposome formation during sonication.

The collapse of a macroscale or two-dimensional lipid monolayer has been investigated extensively^30^,^31^,^32^,^33^. To explain this collapse, researchers developed free-energy models assuming that the mechanical properties of the monolayer (e.g., bending modulus, surface tension, and line tension) are constant throughout the collapse. The local curvatures in the macroscale monolayer are small and therefore the first-order approximation is applied. In addition, the interaction of the folds is neglected. However, the collapse of a lipid-coated nanobubble, i.e., a highly curved monolayer, potentially involves more complicated factors. For example, the large curvature of the nanobubble and its dynamical change during the collapse (Figs 1 and 2) may affect the curvature-dependent elastic properties, and the bending energy estimation requires higher order contributions^34^. Because the folds grow from multiple directions, the lipid molecular interactions may be pronounced in the monolayer–monolayer adhesion when a bilayer forms or in the folds’ fusion^35^. The water vapor pressure in the bubble may not be negligible because it increases when the bubble shrinks and therefore affects the folds’ growth. To our knowledge, it is still challenging to simulate water vapor characteristics in classical MD simulations. Moreover, thermal effects associated with acoustic cavitation may also affect the collapse of lipid monolayers and liposome formation^36^ even though we did not consider them here.

The percentages of vesiculation in the 20 samples were found to be 0%, 10%, 100%, 100% for the 600-, 1200-, 2400-, and 4800-lipid systems, respectively (Fig. 2). This suggests that the 1200-lipid system is metastable, i.e., disks and vesicles can coexist. On the basis of the phenomenological model^4^, we analyzed the vesicular index 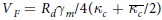, where *R*_*d*_ is the apparent radius of the disk, *γ*_*m*_ is the line tension, *κ*_*b*_ is the bending modulus, and 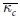 is the saddle-splay modulus. A disk is stable with respect to closure (vesiculation) if *V*_*F*_ < 1; it is metastable if 1 ≤ *V*_*F*_ ≤ 2; and it is unstable if 2 < *V*_*F*_. Assuming 
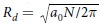
, the vesicular index becomes 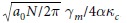, where *α*_0_ is the area per lipid, *N* is the number of lipids in the disk, and 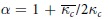. Taking the values *κ*_*c*_ = 16.6 × 10^−20^ J, 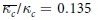, *γ*_*m*_ = 50 pN, and *α*_0_ = 0.64 nm^2^ for the MARTINI DPPC lipids^37^,^38^, *V*_*F*_ is estimated to be 0.55, 0.78, 1.10 and 1.56 for the 600-, 1200-, 2400-, and 4800-lipid systems, respectively. This may be acceptable when considering the actual complicated disk shape (Figs 2 and S2) and the variation in the mechanical properties, within the limits of the CG model and accessible timescales of the current simulations^37^,^38^,^39^. With regards to the 600 dilinoleyl-PC lipid system, its smaller bending modulus^40^ may increase the vesicular index and therefore induce vesiculation (Fig. S3) even though the mechanical properties for the MARTINI dilinoleyl-PC are, to our knowledge, unclear. In future, more comprehensive studies should be conducted to understand the effects of various lipid compositions on bubble collapse and subsequent liposome formation at the molecular scale.

## Conclusion

We observed the formation of a liposome from a lipid-coated nanobubble using CG MD simulations of the DPPC lipid–water systems. A preformed nanobubble coated with a lipid monolayer in water was our model for the aqueous dispersion of phospholipids during sonication. We identified the intermediate stages in the liposome formation from a nanobubble, such as the irregular bubble deformation, the local monolayer buckling and folding and their growth from or to multiple directions, the discoidal membrane formation, and the vesicle formation via a bowl shape. Because the discoidal membrane after the collapse has folds of various numbers and amplitudes, it occasionally develops into a multivesicular shape. Even when the unilamellar vesicle forms, the folds induce the imbalanced number of lipids between the inner and outer monolayers of the resultant vesicle shape. In addition, the saturation of phospholipid tails affects the vesicle formation process by forming a more complicated discoidal membrane shape. Consequently, we concluded that the discoidal membrane shape after the nanobubble collapse can be an important factor governing the vesicular shape during sonication.

## Methods

Lipid–water systems are composed of CG-saturated phosphatidylcholine (PC), which corresponds to PCs with tail lengths of C15-18, i.e., DPPC-like lipids. Water molecules are based on the MARTINI force field^37^,^41^, where the molecules are represented by grouping four heavy atoms into a bead. The initial structure was a preformed nanobubble coated with a lipid monolayer in water. To this end, water beads within a given distance from the center of a cubic liquid water system, which was pre-equilibrated to 323 K and 1 bar, were hollowed out to generate a spherical void region. Then, the void interface was coated by placing lipid molecules where the hydrophilic head groups of the lipid molecules were stuck into the interface. The base system was composed of 600 lipid and 78,501 water CG models. Periodic boundary conditions were used in all directions.

Subsequent to the energy minimization of the initial configuration, constant temperature and volume MD calculations were performed at 323 K to equilibrate the systems for at least 200 ns. The leapfrog algorithm was used for integrating the equations of motion and the time step was set to 20 fs. The bonded and nonbonded interaction settings were the same as in the original paper on the MARTINI force field. The temperatures of the DPPC and the water were kept constant separately using the velocity rescaling method with a 1.0 ps coupling constant^42^. During the constant temperature and volume MD simulation, water beads were spontaneously evaporated to the void region and the lipid nanobubble contained the evaporated water beads (Fig. 1(a)). Note that when the lipid monolayer collapsed or ruptured during the constant volume MD calculation, we changed the system size and restarted the calculation such that the water liquid–vapor interface was fully coated by the CG lipid monolayer. The number density of the CG beads for the 600-lipid system was 7.86 -/nm^3^.

After the constant volume MD calculation, the pressure was relaxed to 1 bar using the Berendsen’s isotropic coupling method with a 2.0 ps coupling constant and a compressibility of 3 × 10^−5^ 1/bar^43^. Twenty different initial configurations taken from every 10 ns of the constant volume MD calculation were used to statistically investigate the liposome formation from the nanobubble. The time scale shown in the manuscript is the simulated time, although, the effective time could be greater^37^,^41^. Note that the target pressure is arbitrary because the MARTINI water CG model does not properly reproduce the characteristics of the pure water liquid–vapor interface^44^. We chose the MARTINI force field in an attempt to maintain the semi-quantitative features for phospholipids while reducing the computational costs to analyze the nanobubble collapse.

To verify the effects of the number of lipids coating the nanobubble, the 1200-, 2400-, and 4800-lipid systems were constructed on the basis of the 600-lipid system, maintaining the water bead/lipid ratio. The preparation of a larger number of lipid systems is arbitrary. The schematic diagram for our approach is shown in Fig. S4. First, the base lipid system is stretched (Stage 1) using the position scaling method of the *deform code* in the GROMACS 4.6.7 software packages^21^,^45^,^46^ until the monolayer is ruptured and a part of the liquid water surface is directly exposed to the water vapor phase^47^. Second, the stretched base system is duplicated so that the ruptured monolayers, i.e., the pore regions, face each other (Stage 2) and constant volume MD simulations are performed to merge the lipid monolayers (Stage 3). Then, the rectangular system is deformed into the cubic system (Stage 4). The number density of the CG beads is 7.86, 7.65, 7.38 and 7.13 -/nm^3^ for the 600-, 1200-, 2400-, and 4800-lipid systems, respectively.

Before we set the density of the initial configuration, we performed a test calculation in which the initial configuration was a nanobubble partly coated with a lipid monolayer. Subjected to a positive pressure, the bubble shape became semi-stable when the liquid–vapor interface was fully coated with the lipid monolayer before the collapse (see Fig. S5). Therefore, to analyze the lipid dynamics effectively, we set the fully lipid-coated bubble as the initial configuration in this study. The apparent area per lipid for the systems are in the range 0.6–0.7 nm^2^, where the coating monolayer is expected in the liquid-expanded phase^30^.

Unsteady and nonequilibrium MD simulations should be performed with great care for various simulation parameters and system sizes. We tested different coupling constants (10 ps and 20 ps for the thermostat and barostat, respectively), different compressibility parameters for the Berendsen’s coupling method (3 × 10^−6^ 1/bar and 3 × 10^−4^ 1/bar, see Fig. S6 in the Supplementary Information), and larger water phase systems (at most 4800 lipids and 1,884,024 water beads). The essential formation dynamics were independent of the parameters and system sizes were tested here. Further, to investigate the impact of the saturation of phospholipid tails, unsaturated PC lipid systems, which were composed of the MARTINI dilinoleyl-PC model, were prepared. We performed CG MD simulations of 10 samples for the 600-lipid system and one sample for the 4800-lipid and a large number of water beads (1,884,024) system.

The MD calculations and the trajectory processing were performed using the GROMACS 4.6.7 software packages^45^,^46^. The in-house Python codes developed with the aid of the MDAnalysis library^48^ were used for the trajectory analysis and all snapshots were rendered using visual molecular dynamics^49^. The in-house Python codes and the input files for the GROMACS are available from the authors upon request.

## Additional Information

**How to cite this article**: Koshiyama, K. and Wada, S. Collapse of a lipid-coated nanobubble and subsequent liposome formation. *Sci. Rep.* **6**, 28164; doi: 10.1038/srep28164 (2016).

## Supplementary Material

Supplementary Information
