# Supplementary material for: Collapse of a lipid-coated nanobubble and subsequent liposome formation
Source: Sci Rep. 2016 Jun 16;6:28164. doi: 10.1038/srep28164 (PMC4910104; doi:10.1038/srep28164)
Supplement: Supplementary Information [file srep28164-s1.pdf]

Supplementary Information: Collapse of a lipid coated nanobubble and subsequent liposome formation

Kenichiro Koshiyama* and Shigeo Wada

Graduate School of Engineering Science, Osaka University, Toyonaka 560-8531, Japan

*koshiyama@me.es.osaka-u.ac.jp

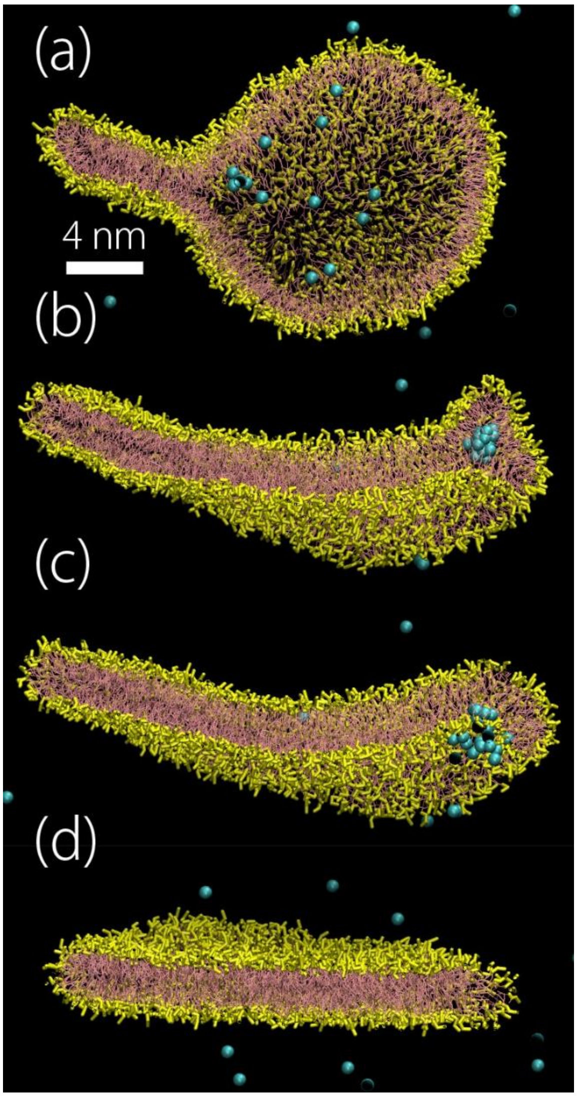

Figure S1. Cutaways of the lipid assembly during the collapse of a lipid nanobubble for the 2400 lipid system at 26, 30, 31, and 50 ns.

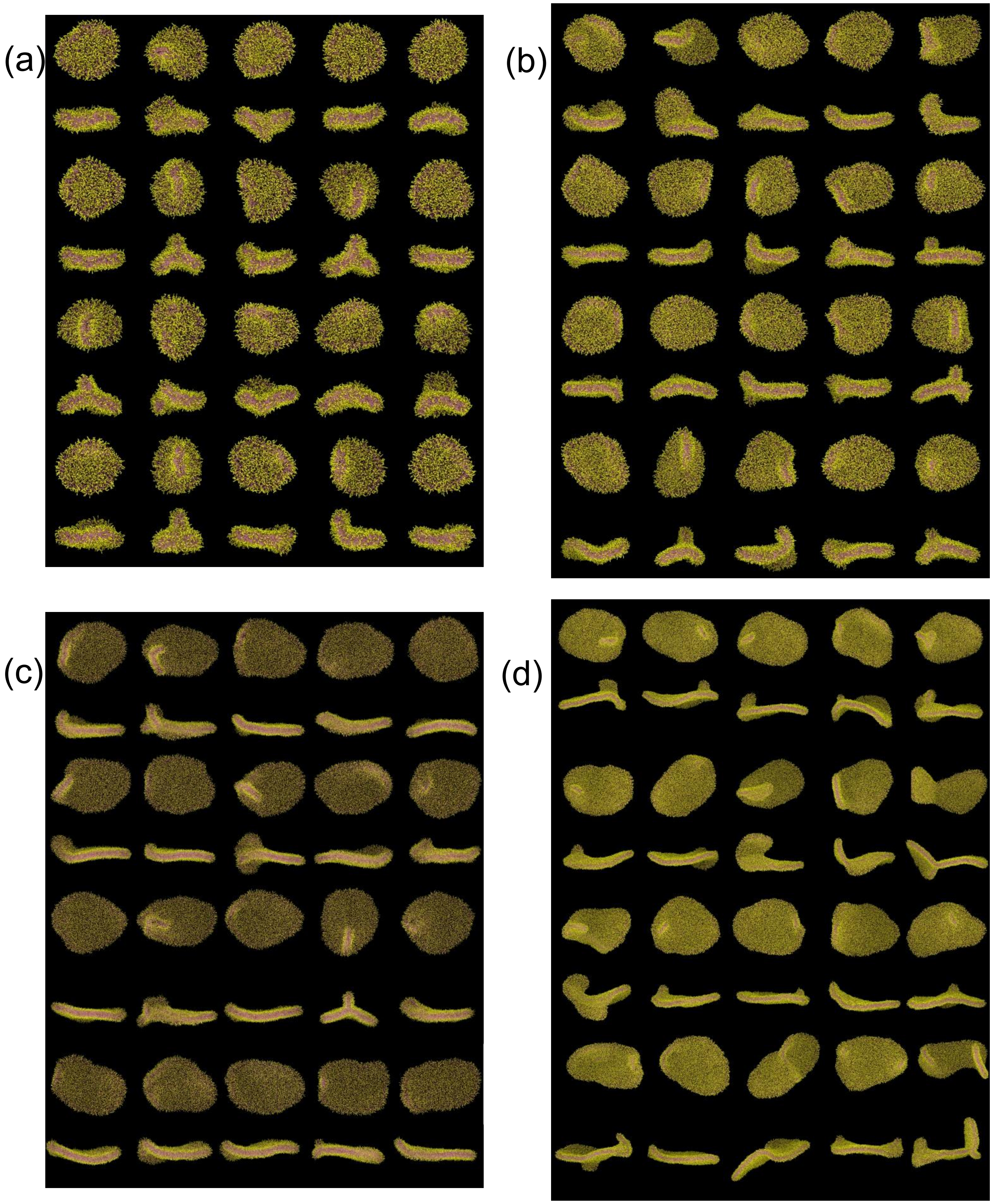

Figure S2. Discoidal lipid assembly just after the nanobubble collapse of 20 samples for 600 (a), 1200 (b), 2400 (c), and 4800 (d) lipid systems. The top and side views are shown for each sample.

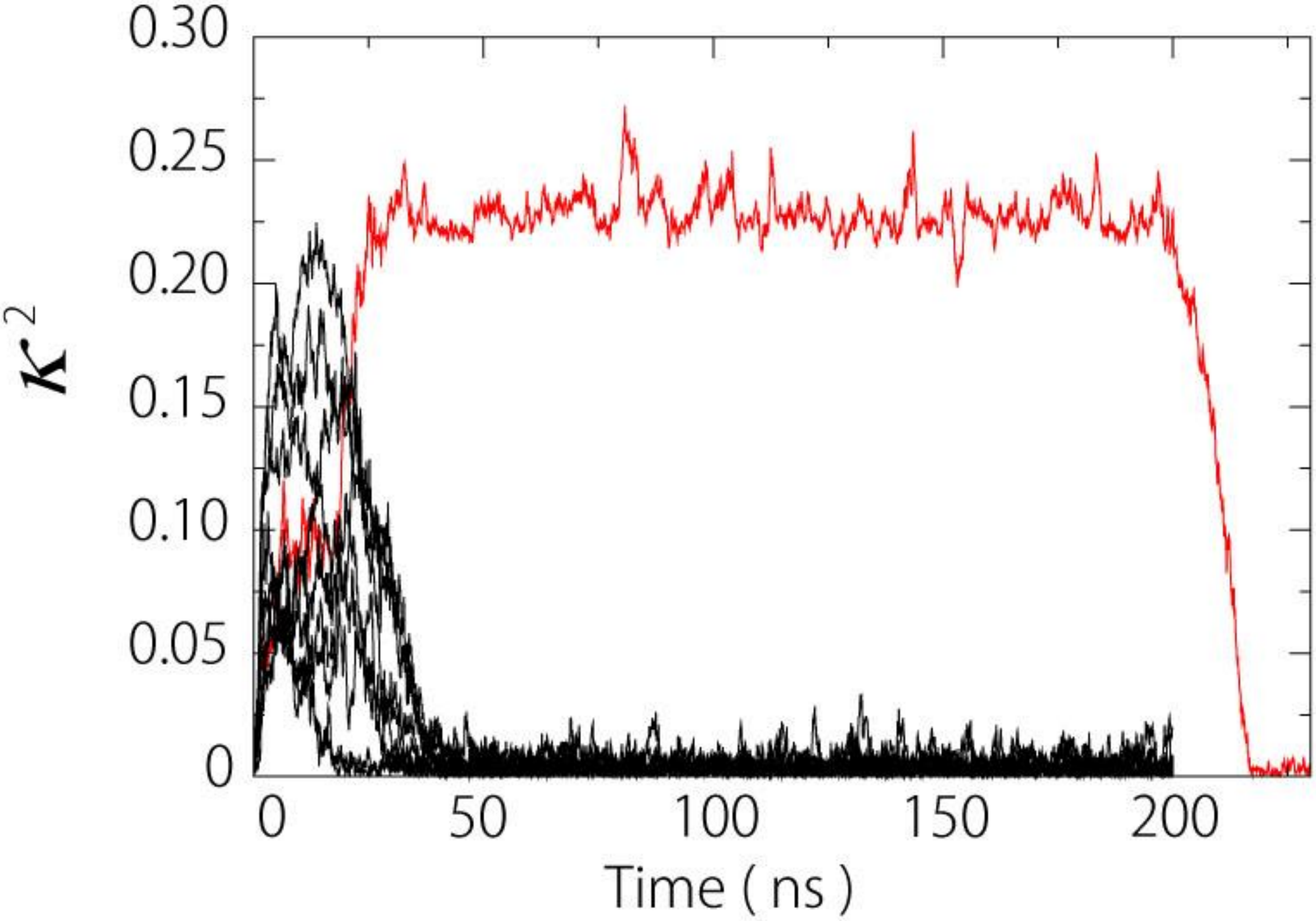

Figure S3. Temporal changes in the relative shape anisotropy κ^2 of 10 samples for the 600 dilinoleyl-PC lipid system. The redline shows the process of liposome formation via an apparent flat discoidal membrane shape.

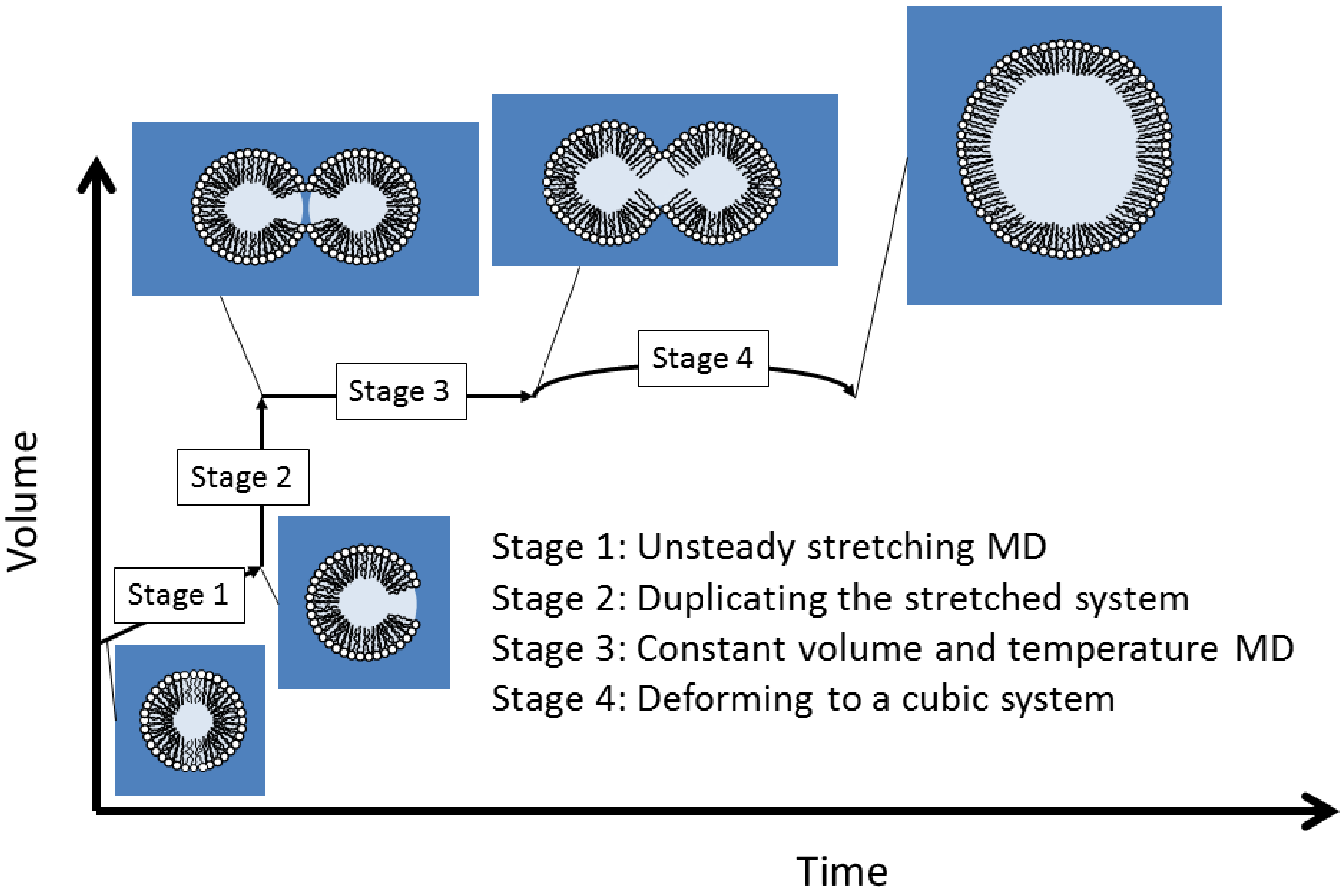

Figure S4. Schematic diagram of preparing a large system from a small system.

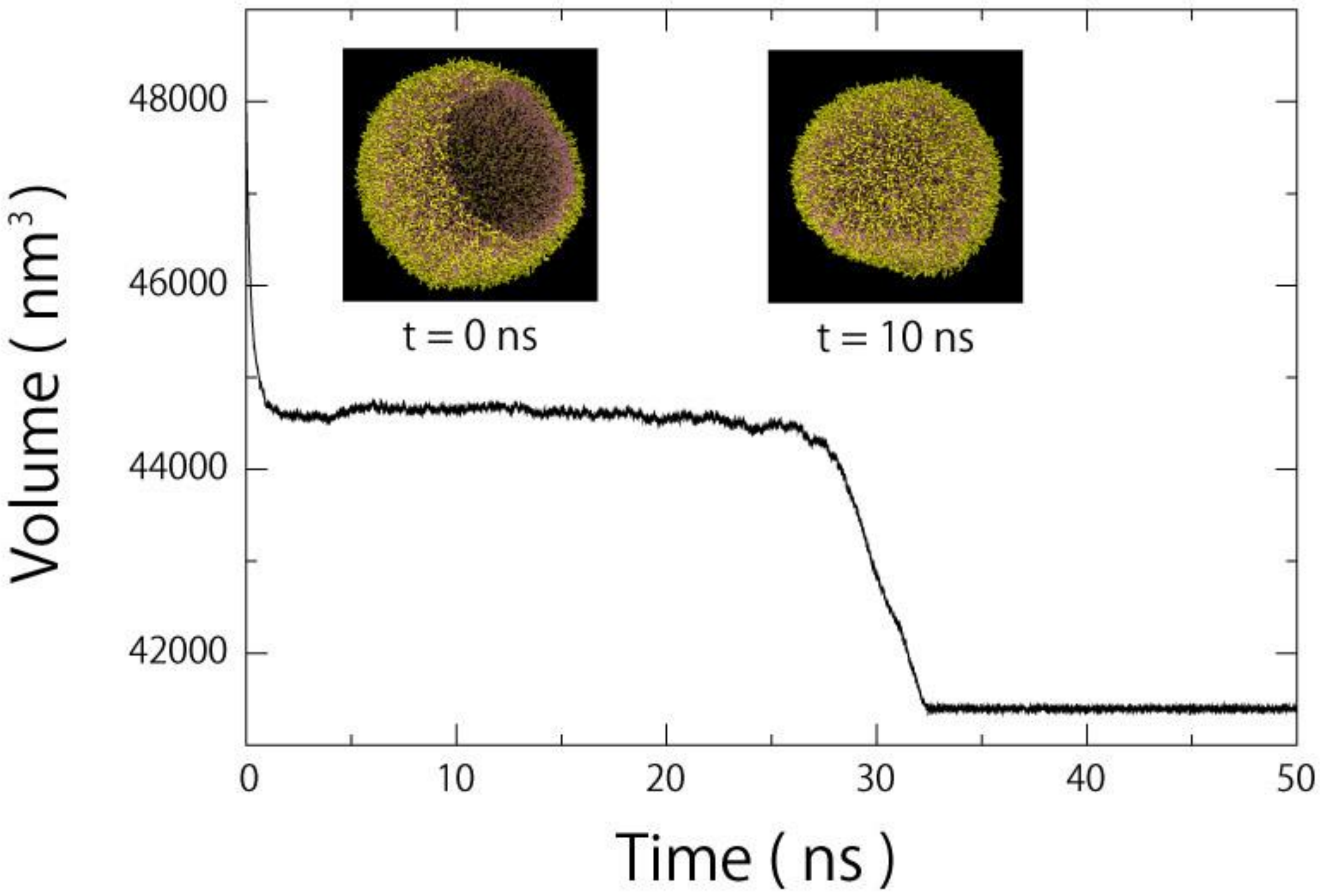

Figure S5. Temporal changes in volume of a lipid nanobubble initially coated by a ruptured monolayer. Insets show the snapshots of the bubble at 0 and 10 ns.

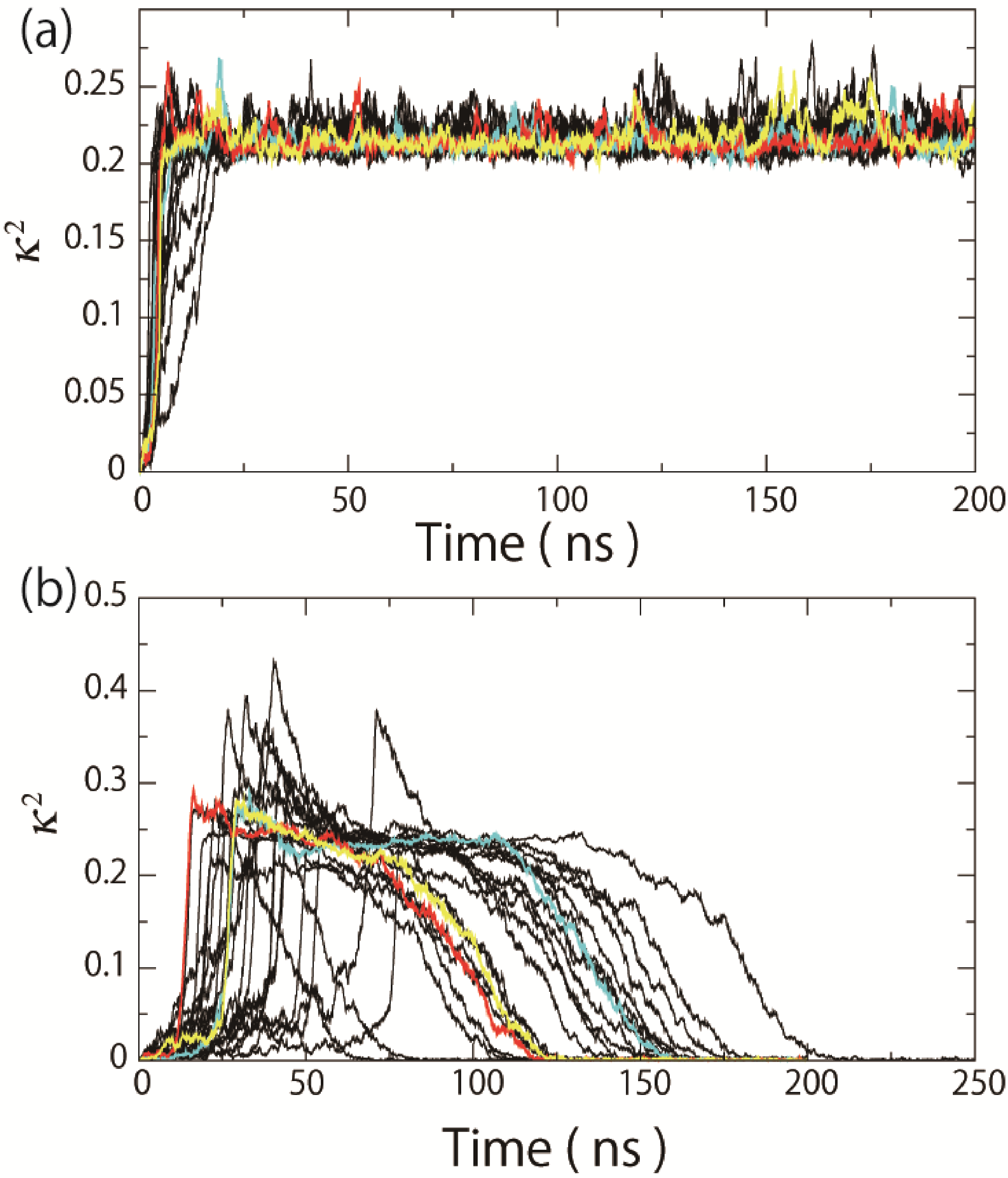

Figure S6. Temporal changes in the relative shape anisotropy κ^2 for the 600- (top) and 2400- (bottom) lipid systems. The cyan line shows the process for the compressibility of 3×10^{-4} 1/bar, the red line for the compressibility of 3×10^{-6} 1/bar, and the yellow line for the temperature coupling constant of 10 ps and the pressure coupling constant of 20 ps. The black lines show the processes of 20 samples for the compressibility of 3×10^{-5} 1/bar.
